# Effects of Total Flavone from* Rhododendron simsii* Planch. Flower on Postischemic Cardiac Dysfunction and Cardiac Remodeling in Rats

**DOI:** 10.1155/2017/5389272

**Published:** 2017-06-08

**Authors:** Xinqi Cheng, Jie Zhang, Zhiwu Chen

**Affiliations:** Department of Pharmacology, Anhui Medical University, Hefei, Anhui 230032, China

## Abstract

This study investigated the effect of total flavone from* Rhododendron simsii* Planch. flower (TFR) on postischemic cardiac dysfunction and ventricular remodeling and was to test the hypothesis that TFR has an antiventricular remodeling effect through inhibition of urotensin-II receptor- (UTR-) mediated activation of RhoA-ROCK pathways. Twenty-four hours after ligation of the left anterior descending coronary artery, male Sprague-Dawley rats were randomized to receive 4-week treatment with saline (model group) or TFR. Compared to the model group, TFR treatment restored cardiac function, attenuated cardiomyocyte hypertrophy, and reduced interstitial fibrosis. Expression levels of several fibrosis-related factors, including alpha-smooth muscle actin, transforming growth factor-beta 1, matrix metalloproteinase-2, and collagen type I, were increased after MI. TFR treatment attenuated the upregulation of these factors, downregulated UTR expression, and markedly diminished the expression of RhoA and ROCK1/2. These results suggested that TFR could improve cardiac function and ameliorate ventricular remodeling through blocking UTR-mediated activation of RhoA-ROCK pathways in myocardial infarction rats.

## 1. Introduction

Progressive cardiac remodeling, which may include myocyte hypertrophy, interstitial fibrosis, and ventricular dilation [1, 2] after myocardial infarction (MI), is a leading cause of morbidity and mortality worldwide. Cardiac fibrosis is characterized by pathological accumulation of extracellular matrix (ECM) with increased expression of fibrosis-related proteins, such as alpha-smooth muscle actin (*α*-SMA) [3], transforming growth factor-beta 1 (TGF-*β*1) [4], matrix metalloproteinase-2 (MMP-2) [5], and collagen type I (collagen I) [6]. In the absence of effective therapies, cardiac fibrosis can lead to ventricular dysfunction and heart failure [7, 8]. Novel approaches for preventing post-MI cardiac fibrosis are greatly needed.

Urotensin-II (U-II) is a small peptide that is expressed widely in the cardiovascular system. U-II can induce cardiomyocyte hypertrophy [9]. Upregulation of the U-II receptor (UTR) may worsen cardiac hypertrophy, accelerate cardiac fibrosis, and increase collagen synthesis [10]. Rho-kinase (ROCK), which has two isoforms, ROCK1 and ROCK2, is an important downstream effector of RhoA. The RhoA-ROCK pathway plays an important role and is a novel therapeutic target for cardiovascular diseases [11]. Activation of the RhoA/ROCK pathway can induce activation of hypertrophy-related genes [12, 13]. Activation of RhoA is involved in post-MI cardiac fibrosis [14]. Several studies have demonstrated a relationship between U-II/UTR and the RhoA-ROCK pathway in the migration of vascular smooth muscle cells and acute myocardial injury [15, 16]. Our recent study demonstrated that inhibition of the UTR-RhoA-ROCK pathway exerts a potent protective effect on MI in rats [16]. However, the role of the UTR-RhoA-ROCK pathway in cardiac remodeling has not been determined.

*Rhododendron simsii* Planch. flower is a Chinese herbal medicine that has an important traditional use in the treatment of bronchitis, pain, and coronary heart disease which is associated with anti-inflammatory, analgesic, and cardioprotective effects. Total flavones from* Rhododendron simsii* Planch. flower (TFR), an effective part of this flower, are comprised of flavones such as hyperin, rutin, quercetin, and other flavonoids [17, 18]. It was reported that some flavonoids have potential antioxidant, anti-inflammatory, and antifibrotic effects, with the latter being elicited through modulation of the cardiac U-II/UTR pathway [19] and inhibition of ROCK activity [20, 21]. Our previous studies found that TFR protects the heart against acute MI injury in rabbits [22] and rats [23], and TFR also has a protective effect on cultured rat cardiomyocytes anoxia/reoxygenation damage via inhibition of ROCK1/2 [24]. Considering the aforementioned studies, we hypothesized that UTR-RhoA-ROCK pathway plays an important role in the cardiac remodeling process and TFR may exert a potential antifibrotic effect via inhibiting the UTR-RhoA-ROCK pathway. Hence, the present study was designed to investigate whether TFR could inhibit ventricular remodeling after coronary artery ligation-induced MI in rats and to elucidate the potential involvement of the UTR-RhoA-ROCK pathway in this process.

## 2. Materials and Methods

### 2.1. Drugs and Reagents

TFR (flavone contents > 85%) was provided by Hefei Heyuan Medicine Technology Co., Ltd. (Hefei, China). Primary antibodies used in this study included antibodies against TGF-*β*1 (sc-52893; Santa Cruz Biotechnology, Santa Cruz, CA, USA), glyceraldehyde-3-phosphate dehydrogenase (GAPDH, sc-25778; Santa Cruz Biotechnology), *α*-SMA (14395-1-AP; Proteintech Group, Chicago, IL, USA), MMP-2 (WL01579a; Wangleibio Co., Shenyang, China), collagen I (WL0088; Wangleibio Co.), UTR (ABIN220283; Antibodies Online Inc., Atlanta, GA, USA), and ROCK1/2 (Nanjing Enogene Biological Co., Nanjing, China).

### 2.2. Rat MI Model

Male Sprague-Dawley (SD) rats weighing 180–220 g were purchased from the Experimental Animal Center of Anhui Medical University. Rats were housed at 22 ± 2°C and a relative humidity of 40 ± 5% under a 12-hour light/dark cycle. Left thoracotomy was performed through the fourth intercostal space, and the pericardium was opened. The left anterior descending coronary artery (LAD) was permanently ligated near its origin with a 6-0 Prolene suture. Evidence of MI was confirmed by electrocardiogram (ECG) S-T segment elevation and appearance of the Q wave in lead II. Rats in the sham group underwent the same surgical procedure, except that the suture under the LAD was not tied. All experimental procedures were approved by the Ethics Committee of Animal Research of the University Health Science Center. The investigation conformed to the* Guide for the Care and Use of Laboratory Animals* published by the US National Institutes of Health (NIH Publication, 8th Edition, 2011).

### 2.3. Protocols and Drug Administration [24]

Animals were randomly divided into the following 6 groups: sham group, model group, 4 mg/kg amlodipine group, 30 mg/kg TFR group, 60 mg/kg TFR group, and 120 mg/kg TFR group. The sham group and model group received equal volumes of 0.9% physiological saline. All drugs, including physiological saline, were orally administered once daily for 4 weeks immediately after MI.

### 2.4. Echocardiography [25]

Blood pressure was measured by tail-cuff plethysmography (BP-98A; Softron, Tokyo, Japan). At the surgical procedure and the end of the experiment, rats under anesthesia (1% pentobarbital) were examined by two-dimensional transthoracic echocardiography in a standard setting by using a 30 MHz high-frequency scan head (VisualSonics Vevo 770; VisualSonics Inc., Toronto, ON, Canada). The left ventricular (LV) internal dimension at diastole (LVIDd) and at systole (LVIDs) and left ventricular posterior wall thickness at diastole (LVPWd) and at systole (LVPWs) were measured, respectively, with the calculation of the LV fractional shortening (LVFS) and ejection fraction (LVEF). All parameters were measured over three consecutive cardiac cycles by one experienced echocardiographer who was blinded to the treatment.

### 2.5. Morphological, Histological, and Immunohistochemical Analyses

After echocardiography was performed, the heart was rapidly harvested and weighed. Heart weight to body weight index (HWI) was determined. Tissues between the point of ligation and the apex of the heart were immediately cut into 2 mm thick transverse sections and immersed in 10% neutral buffered formalin for 24 hours. Serial LV cross sections (6 *μ*m in thickness) were deparaffinized and stained with hematoxylin/eosin. Remaining tissue samples were flash-frozen in liquid nitrogen and stored at −80°C for further assays.

### 2.6. Picrosirius Red Staining

Tissue sections were stained with Picrosirius Red. Interstitial fibrosis was quantified by the ImageJ analysis software (NIH, Bethesda, MD, USA). The positively red area was expressed as a percentage of the total area.

### 2.7. Western Blot Analysis

Protein was extracted from heart tissues of the infarct border zone. A BCA protein assay kit (Beyotime, Nantong, China) was used to estimate protein concentration. Aliquots of 30 *μ*g of protein from each sample were loaded and electrophoresed in 8% or 12% SDS-polyacrylamide gel and transferred to a polyvinylidene difluoride membrane (Thermo Scientific). Membranes were blocked with 5% nonfat dry milk and probed with antibodies for UTR, ROCK1/2, TGF-*β*1, *α*-SMA, MMP-2, and collagen I overnight at 4°C. Blots were washed thrice and incubated with horseradish peroxidase-conjugated secondary antibody (Abbkine, Inc., USA) for 2 hours. GAPDH was used as the internal control. Immunocomplexes were visualized using ECL Plus (Thermo Scientific). Densities of bands were also quantified by using the ImageJ analysis software.

### 2.8. Measurement of RhoA Activity

Expression of GTP-RhoA protein was measured to indicate RhoA activity. GTP-RhoA was assessed by using commercially available kits from Cytoskeleton (BK 036-S) following the manufacturer's instructions. Lysis buffer containing protease inhibitors was used to harvest heart tissues. To detect the activity of RhoA, equal volumes of clarified lysates were incubated with 50 *μ*g of the rhotekin-RBD beads (Rho-GTPase binding domain) at 4°C for 1 hour. The beads were washed once with 500 *μ*l each of Wash Buffer. Beads were then briefly boiled in 2x Laemmli buffer. Active GTP-RhoA and whole cell lysates were subjected to be analyzed by SDS-PAGE and western blot analysis.

### 2.9. Statistical Analysis

All data are presented as the mean ± standard error of the mean (SEM). Differences were analyzed by one-way ANOVA for multiple groups, followed by Tukey's multiple comparison tests with GraphPad Prism 5.0 software (GraphPad Prism, CA, USA). Survival analysis was performed by the Kaplan–Meier method. Between-group differences in survival were tested by the log-rank test. A value of *P* < 0.05 was considered statistically significant.

## 3. Results

### 3.1. Effects of TFR on Rat Survival Rates

Four weeks after surgery, BP was decreased and HR was increased at baseline in saline-treated MI animals compared with sham-operated animals (data not shown). Ninety-seven rats underwent surgery, either with (*n* = 91) or without (*n* = 6, sham group) LAD ligation. Sixty rats (60/97, 61.9%) that had received LAD ligation died within 24 hours after surgery. At the end of the experiment, the survival rate was significantly higher in TFR 30, 60, and 120 mg/kg groups than in the model group. Similar to TFR, 4 mg/kg amlodipine also enhanced the survival rate (data not shown).

### 3.2. Effects of TFR on MI-Induced Change of Cardiac Weight Index

**Figure 1:**
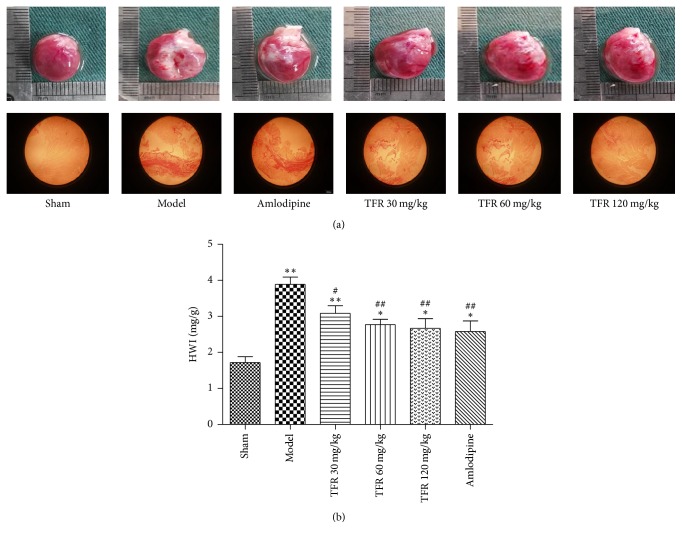
Effects of TFR on the cardiac weight index (heart weight to body weight index, HWI) after MI in rats. (a) The photographs of representative hearts from different groups. (b) Effects of TFR on HWI. Data are presented as the mean ± SEM. ^*∗*^*P* < 0.05 and ^*∗∗*^*P* < 0.01, compared with the sham group; ^#^*P* < 0.05 and ^##^*P* < 0.01, compared with the model group.

In contrast with the sham group, hearts in the model group exhibited obvious infarction, with some evidence of myocardial fibrosis with a pale color and thinner LV wall below the site of ligation. These abnormalities were alleviated in the hearts from TFR-treated rats (Figure 1(a)). The cardiac weight index (HWI), which was increased in the model group, was significantly attenuated by treatment with TFR 30, 60, and 120 mg/kg (*P* < 0.01 or *P* < 0.05). And 4 mg/kg amlodipine had a similar effect (Figure 1(b)).

### 3.3. Effects of TFR on MI-Induced Cardiac Dysfunction

**Figure 2:**
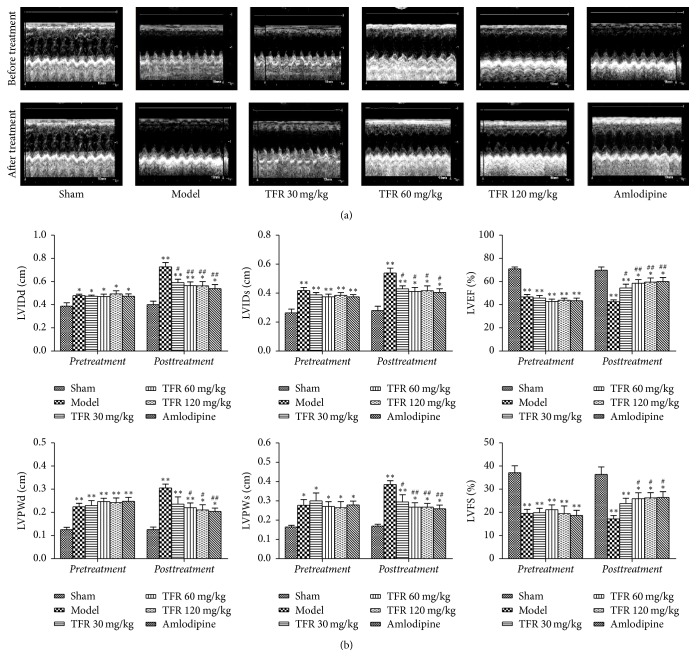
Effects of TFR on the cardiac function after MI in rats. (a) M-mode echocardiographic images for each group before and after drug administration. (b) Effect of TFR on parameters of cardiac function. LVIDd: left ventricular internal dimension at diastole; LVIDs: left ventricular internal dimension at systole; LVFS: left ventricular fractional shortening; LVEF: left ventricular ejection fraction; LVPWd: left ventricular posterior wall thickness at diastole; LVPWs: left ventricular posterior wall thickness at systole. Data are presented as the mean ± SEM. ^*∗*^*P* < 0.05 and ^*∗∗*^*P* < 0.01, compared with the sham group; ^#^*P* < 0.05 and ^##^*P* < 0.01, compared with the model group.

Examination of echocardiograms showed that rat cardiac function was remarkably deteriorated in the model group compared to that in the sham group, as evidenced by increased LVIDs, LVIDd, LVPWs, and LVPWd and decreased LVFS and LVEF. Treatment with TFR 30, 60, and 120 mg/kg or amlodipine 4 mg/kg significantly inhibited these changes in cardiac function indices (*P* < 0.01 or *P* < 0.05), suggesting that TFR or amlodipine could ameliorate the myocardial dysfunction (Figure 2).

### 3.4. Effects of TFR on MI-Induced Myocyte Hypertrophy and Cardiac Fibrosis

**Figure 3:**
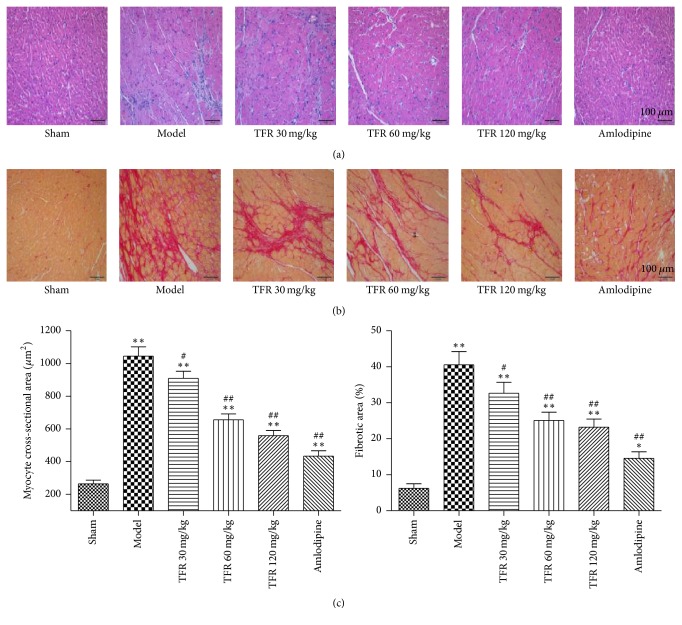
Effects of TFR on myocyte hypertrophy and cardiac fibrosis in rats with ventricular remodeling after MI. (a) HE staining of myocardium in the noninfarcted area. (b) Interstitial fibrosis in the noninfarcted area was evaluated by picric acid Sirius Red staining. (c) Quantitative analysis of cardiomyocyte cross-sectional area and ratio of fibrotic area (red) to total area. Data are presented as the mean ± SEM. ^*∗*^*P* < 0.05 and ^*∗∗*^*P* < 0.01, compared with the sham group; ^#^*P* < 0.05 and ^##^*P* < 0.01, compared with the model group.

The cardiomyocyte cross-sectional area was significantly larger in the model group than in the sham group (*P* < 0.01, Figure 3). The average cross-sectional area of cardiomyocytes was significantly decreased in TFR 30, 60, and 120 mg/kg groups (*P* < 0.01 or *P* < 0.05) and in 4 mg/kg amlodipine group (*P* < 0.01) compared to that in the model group. Examination of Picrosirius Red-stained sections revealed a significantly higher extent of interstitial fibrosis in the LV wall from the model group compared to the sham group. Administration of TFR 30, 60, and 120 mg/kg or amlodipine 4 mg/kg markedly reduced the extent of fibrotic deposits in the LV wall (*P* < 0.01 or *P* < 0.05).

### 3.5. Effects of TFR on MI-Induced Increase of Fibrosis-Related Factors Expressions

**Figure 4:**
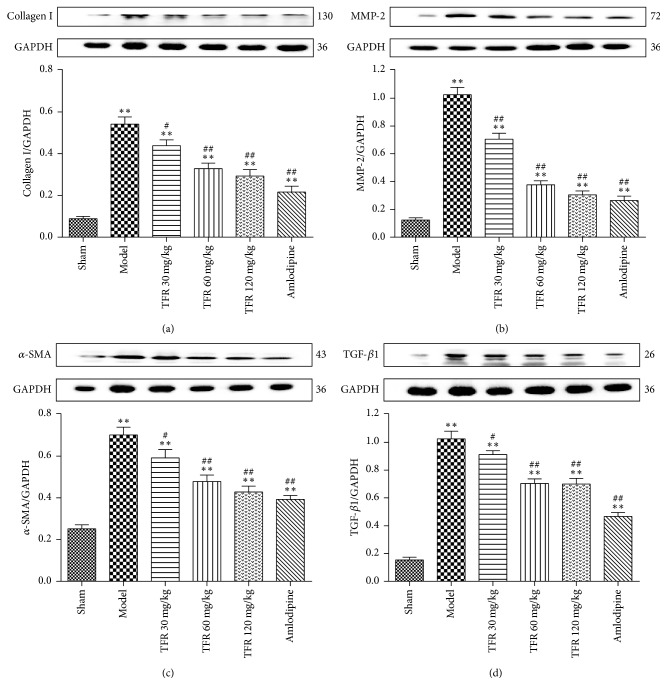
Effects of TFR on expressions of fibrosis-related factors proteins in the noninfarcted cardiac area. (a–d) The western blot analysis of collagen type I (collagen I), matrix metalloproteinase-2 (MMP-2), *α*-smooth muscle actin (*α*-SMA), and transforming growth factor-*β*1 (TGF-*β*1). Data are presented as the mean ± SEM. ^*∗∗*^*P* < 0.01, compared with the sham group; ^#^*P* < 0.05 and ^##^*P* < 0.01, compared with the model group.

As shown in Figure 4, expression levels of fibrosis-related factors, including *α*-SMA, TGF-*β*1, MMP-2, and collagen I, were used to ascertain the degree of myocardial fibrosis. Treatment with TFR 30, 60, and 120 mg/kg significantly inhibited expression levels of *α*-SMA, TGF-*β*1, MMP2, and collagen I (*P* < 0.05, *P* < 0.01), which had been significantly upregulated after MI, respectively (*P* < 0.01). 4 mg/kg amlodipine had a similar effect on the expressions of *α*-SMA, TGF-*β*1, MMP-2, and collagen I.

### 3.6. Effects of TFR on MI-Induced Activation of UTR-RhoA/ROCK Pathway

**Figure 5:**
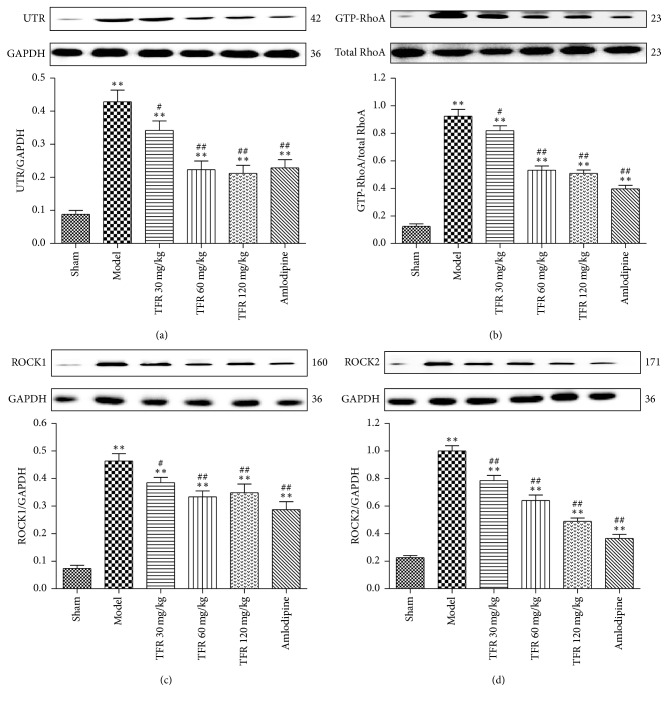
Effects of TFR on the activation of UTR-RhoA/ROCK pathway in the noninfarcted cardiac area. (a–d) The western blot analysis of U-II receptor (UTR), GTP-RhoA (GTP-binding RhoA), ROCK1, and ROCK2. Data are presented as the mean ± SEM. ^*∗∗*^*P* < 0.01, compared with the Sham group; ^#^*P* < 0.05 and ^##^*P* < 0.01, compared with the Model group.

To investigate probable action mechanisms of TFR on MI-induced cardiac dysfunction and cardiac remodeling, UTR, GTP-RhoA, and ROCK1/2 protein expressions in the infarct border zone were analyzed. As shown in Figure 5, UTR, GTP-RhoA, ROCK1, and ROCK2 protein expressions were significantly upregulated after MI (*P* < 0.01) compared with those in the sham group. By contrast, treatment with TFR 30, 60, and 120 mg/kg could significantly inhibit MI-induced upregulation of UTR, GTP-RhoA, ROCK1, and ROCK2 protein expressions (*P* < 0.01 or *P* < 0.05). Administration of amlodipine 4 mg/kg had a similar effect to that observed in the TFR-treated groups.

## 4. Discussion

Ventricular remodeling after acute MI results from infarct expansion followed by hypertrophy. In the present study, a MI-induced ventricular remodeling model was established in rats, and this model exhibits LV enlargement, cardiac dysfunction, and high mortality rates up to 4 weeks after MI. In this rat model, HWI (a heart weight index) and the transverse area of cardiomyocytes are the main indicators of the degree of cardiac hypertrophy [26, 27]. Our results showed that HWI and average cross-sectional area of cardiomyocytes significantly increased in the model group, whereas TFR treatment significantly improved changes of the two indicators of cardiac hypertrophy. Our result also showed that TFR treatment could enhance rat mortality rates.

Interstitial fibrosis represents a characteristic pathological remodeling process after MI. The fibrotic process involves transdifferentiation of cardiac fibroblasts into myofibroblasts, and it is a major determinant of the progressive deterioration of ventricular function after MI [28]. In the present study, the examination of Picrosirius Red-stained sections revealed that significant interstitial fibrosis occurred in the LV wall in the model group. By using echocardiograms, MI-induced cardiac dysfunction was examined. The result showed that rat cardiac function deterioration was indicated by increased LVIDs, LVIDd, LVPWs, and LVPWd and decreased LVFS and LVEF. However, this structural and functional remodeling in rat LV was improved by the treatment with TFR at the range of 30~120 mg/kg.

Activation of TGF-*β*1 plays an important role in the pathogenesis of post-MI-induced LV fibrosis and remodeling [29]. Inflammation after MI may lead to an increase of TGF-*β*1 expression, thereby accelerating myocardial fibrosis by modulating the conversion of fibroblasts to myofibroblasts and the subsequent production of ECM [30]. These effects could alter the cardiac electrophysiology, decrease ventricular compliance, impair heart pumping and distensibility, and contribute to cardiac diastolic and systolic dysfunctions [31]. The myofibroblast, which is characterized by *α*-SMA expression, plays a major role in the pathogenesis of cardiac fibrosis by secreting cytokines, growth factors, and ECM proteins [32]. Hence, *α*-SMA is an important index for myofibroblast and cardiac fibrosis. MMP-2 and collagen I levels are also two biomarkers of interstitial fibrosis after MI [33, 34]. Our results demonstrated that MI induced significant increases of TGF-*β*1, *α*-SMA, MMP-2, and collagen I in the rat myocardium. However, treatment with TFR could markedly inhibit these increases. Taken together, these results suggest that TFR exerts an antifibrotic effect not only through attenuating TGF-*β*1 production in the myocardium, but also through inhibiting myofibroblast differentiation and ECM production.

Both the urotensinergic system and the RhoA-ROCK pathway play crucial roles in cardiovascular diseases. U-II and UTR could accelerate cardiomyocyte hypertrophy [9, 10]. Activation of RhoA-ROCK pathway is also involved in MI-induced cardiac remodeling [12–14]. A previous study reported that the beneficial effects of the ROCK inhibitor fasudil are accompanied by suppression of cardiomyocyte hypertrophy and interstitial fibrosis [35]. The different functions of ROCK1 and ROCK2 in the pathogenesis of cardiovascular disease have been well documented [36]. After classical activation by GTP-RhoA, ROCK1 mediates the activation of transcription factors, leading to upregulation of TGF-*β*1 and activation of adjacent cardiac fibroblasts, which could explain the observed fibrosis [37]. In contrast to the proposed role of ROCK1 in cardiac fibrosis, ROCK2 was shown to be an important player in cardiac hypertrophy [38]. Our recent study demonstrated that inhibition of the UTR-RhoA-ROCK pathway exerts a potent protective effect on acute MI in rats [16]. But the role of the UTR-RhoA-ROCK pathway in cardiac remodeling is unclear. The present study indicated that UTR, GTP-RhoA, ROCK1, and ROCK2 protein expressions were significantly upregulated after MI in rats, suggesting that activation of UTR-RhoA-ROCK pathway occurs during chronic cardiac ischemic and remodeling processes.

Our results also showed that treatment with TFR at the range of 30~120 mg/kg could significantly inhibit MI-induced upregulation of UTR, GTP-RhoA, ROCK1, and ROCK2 protein expressions. These, at least in part, show that the antiremodeling effects of TFR may be associated with downregulation of UTR and subsequent inhibition of the RhoA-ROCK signaling pathway.

In conclusion, activation of the UTR-RhoA-ROCK pathway occurs in chronic ischemic-induced cardiac remodeling. Administration of TFR could ameliorate post-MI-induced cardiac dysfunction and remodeling in rats, and these effects were associated with the inhibition of UTR-RhoA-ROCK pathways.

## Abbreviations

TFR: Total flavones from* Rhododendron simsii* Planch. flower
MI: Myocardial infarction
UTR: Urotensin-II receptor
ROCK: Rho-associated coiled-coil forming protein kinase
*α*-SMA: Alpha-smooth muscle actin
matrix TGF-*β*1: Transforming growth factor-beta 1
MMP-2: Metalloproteinase-2.
